# TMEM44 as a Novel Prognostic Marker for Kidney Renal Clear Cell Carcinoma is Associated with Tumor Invasion, Migration and Immune Infiltration

**DOI:** 10.1007/s10528-023-10466-x

**Published:** 2023-08-10

**Authors:** Jie Tian, Liang Sun, Lisong Wan, Haibin Zou, Jitao Chen, Fei Liu

**Affiliations:** 1 Department of Urology, The Second Affiliated Hospital of Nanchang University, Nanchang, China; 2 Department of Hepatobiliary and Pancreatic Surgery, The Second Affiliated Hospital of Nanchang University, Nanchang, China; 3 Department of Organ Transplantation, Jiangxi Provincial People’s Hospital, Nanchang, China; 4 Trauma Center, Shangrao People’s Hospital, Shangrao, China

**Keywords:** Kidney clear cell carcinoma, TMEM44, Prognostic factor, Transmembrane proteins family, Immune Infiltration

## Abstract

Transmembrane (TMEM) proteins are integral membrane proteins that traverse biological membranes. Several members of the TMEM family have been linked to the development and progression of various tumors. However, the specific role and mechanism of TMEM44 in tumor biology remain largely unexplored. In this study, we initially conducted an extensive analysis using the TCGA database to investigate the expression patterns and survival associations of TMEM44 across various human tumors. Subsequently, we focused on KIRC and found a significant correlation between TMEM44 expression and this particular cancer type. To validate our findings, we performed western blot and quantitative polymerase chain reaction (qPCR) assays to confirm the expression levels of TMEM44 in KIRC. Following this, we employed a series of functional assays, including CCK8 viability assay, EDU incorporation assay, wound healing assay, and transwell migration assay, to investigate the biological role of TMEM44 in KIRC. We observed a significant upregulation of TMEM44 expression in KIRC, indicating its potential involvement in the pathogenesis of this cancer. We intervened in the expression of TMEM44 in KIRC cells and found significant inhibitory effects on cell proliferation, migration, and invasion in KIRC cells. Furthermore, our findings indicated that TMEM44 could serve as an independent prognostic factor in KIRC, highlighting its potential clinical significance. Consequently, TMEM44 holds promise as both a prognostic biomarker and a prospective therapeutic target for KIRC.

**Supplementary Information:**

The online version contains supplementary material available at 10.1007/s10528-023-10466-x.

## Introduction

Transmembrane proteins (TMEMs) are found throughout biological membranes and can facilitate the transport of substances across the cell membrane^(Schmit and Michiels 2018)^. TMEMs encompass both α-helical and β-barrel proteins in terms of their structure^(Vinothkumar and Henderson 2010)^. Additionally, TMEMs can be categorized based on their topology, which refers to the positioning of the N-terminal and C-terminal domains^(Heijne 2006)^.

TMEMs play crucial roles in regulating intercellular ion transport, neuronal excitation, and smooth muscle contraction^(Zhao et al. 2017)^. They are present in various cell types and have significant physiological functions. For example, TMEM165 is associated with protein hypoglycosylation^(Foulquier et al. 2012)^. TMEM16A is linked to smooth muscle contraction and cerebral atherosclerosis^(Thomas-Gatewood et al. 2011)^. Several family members have been implicated in tumor development. Knockdown of TMEM45B notably inhibits the proliferation, migration, invasion, and epithelial-mesenchymal transition (EMT) phenotype of gastric cancer cells^(Shen et al. 2018)^. TMEM205 improves the prognosis of hepatocellular carcinoma patients by reducing the presence of immunosuppressive cells (M2 macrophages and Treg) and promoting the infiltration of CD8 + T cells in the tumor microenvironment^(Rao et al. 2020)^. TMEM106C contributes to the malignant characteristics and poor prognosis of hepatocellular carcinoma^(Duan et al. 2021)^. However, research on TMEM44 is limited to gastric cancer and glioma, specifically focusing on TMEM44 antisense RNA^(Zhou et al. 2022; Bian et al. 2021)^. Consequently, there is a knowledge gap regarding TMEM44, prompting us to investigate its potential significance across various tumors through a pan-cancer analysis, with a specific emphasis on kidney clear cell carcinoma (KIRC).

Hence, the objective of this study was to assess the significance of TMEM44 in KIRC and determine its potential influence on the proliferation, migration, and invasion of KIRC cells.

## Materials and Methods

### Expression and Survival Analysis of TMEM44 in Pan-Cancer

Data for human tumors were obtained from TCGA (https://portal.gdc.cancer.gov/). We performed an analysis of TMEM44 gene mutations in pan-cancer using cBioPortal (https://www.cbioportal.org/datasets). The expression of TMEM44 in tumors was assessed using TIMER (http://timer.cistrome.org/), and the gene expression levels were reported as log2 TPM values.

To examine the relationship between TMEM44 expression and survival in pan-cancer, we employed the Cox proportional risk model and conducted Kaplan-Meier analysis. The analysis included Overall Survival (OS), Disease-Specific Survival (DSS), and Progression-Free Survival (PFS) as outcomes of interest.

### Independent Prognostic and Clinical Correlation Analysis and Construction of Nomogram

First, we analyzed the relationship between TMEM44 expression and the clinical characteristics of KIRC. Meanwhile, Additionally, we performed an independent prognostic analysis that incorporated the clinical characteristics of TCGA-KIRC and TMEM44. Furthermore, to provide further evaluation for individual patients, we developed statistical prediction models using Nomograms based on both patient clinical data and TMEM44 expression.

### Tissue Specimens and Immunohistochemical Staining

A total of 20 pairs of human KIRC tissues and adjacent paraneoplastic tissues were obtained from the Department of Urology, Second Affiliated Hospital of Nanchang University. The tissues were processed through paraffin embedding, sectioning, dewaxing, and hydration. The primary antibody was incubated overnight at a dilution of 1:100, followed by secondary antibody labeling for 30 min. Subsequently, the tissues were stained and photographed.

To quantify the expression differences between KIRC tissues and paraneoplastic tissues, the average optical density values of the images were calculated using ImageJ software. These values were then used to determine the relative optical density scores, enabling a comparison of TMEM44 expression levels between the KIRC tissues and the adjacent paraneoplastic tissues.

### Cell Culture and Cell Transfection

The cell lines 786-O, ACHN, 769-P, and HK-2 were obtained from the cell bank of Shanghai Institutes for Biological Sciences, Chinese Academy of Sciences. Specifically, 786-O and 769-P cells were cultured using 1640 medium (Solarbio, Beijing, China), while ACHN and HK-2 cells were treated with high-sugar DMEM supplemented with 10% fetal bovine serum (bio Industries, bett-haemek; Israel) and 100 µg/ml streptomycin and 100 U/ml penicillin (Solarbio, Beijing, China). All cell lines were maintained at 37 °C with 5% CO2.

For the interference sequence targeting TMEM44 and the corresponding negative control, the sequences were designed by Gemma PharmaTech (Shanghai, China) (Table S1). Prior to transfection, cells were seeded into six-well plates and allowed to reach 70-80% confluency in each well. Transfection was performed using Lipofectamine 3000 reagent (Invitrogen) following the manufacturer’s instructions. Cells were collected 72 h post-transfection for subsequent experiments.

### Quantitative Real-Time Polymerase Chain Reaction (qRT-PCR) Analysis

RNA extraction from tissues and cells was performed using the Trizol kit (Invitrogen). Subsequently, cDNA synthesis was carried out using the Reverse Transcription kit (Takara), with GAPDH serving as the internal control. The primer sequences used are provided in Table 1.

**Table 1:** Univariate and Multivariate analysis in KIRC

Characteristics	Total(N)	Univariate analysis		Multivariate analysis	
		Hazard ratio (95% CI)	P value	Hazard ratio (95% CI)	P value
**TMEM44**	539				
Low	269	Reference			
High	270	2.001 (1.469–2.728)	**< 0.001**	2.662 (1.706–4.154)	**< 0.001**
**T**	539				
T1	278	Reference			
T2	71	1.515 (0.908–2.526)	0.112	0.190 (0.036–1.012)	0.052
T3	179	3.354 (2.373–4.742)	**< 0.001**	0.800 (0.206–3.113)	0.748
T4	11	10.829 (5.467–21.451)	**< 0.001**	0.903 (0.205–3.975)	0.892
**N**	257				
N0	241	Reference			
N1	16	3.453 (1.832–6.508)	**< 0.001**	1.577 (0.583–4.262)	0.369
**M**	506				
M0	428	Reference			
M1	78	4.389 (3.212–5.999)	**< 0.001**	0.756 (0.072–7.959)	0.816
**Stage**	536				
Stage I	272	Reference			
Stage II	59	1.207 (0.650–2.241)	0.551	5.232 (0.802–34.140)	0.084
Stage III	123	2.705 (1.800-4.064)	**< 0.001**	2.613 (0.616–11.083)	0.193
Stage IV	82	6.692 (4.566–9.808)	**< 0.001**	12.773 (0.988-165.079)	0.051
**Gender**	539				
Female	186	Reference			
Male	353	0.930 (0.682–1.268)	0.648		
**Age**	539				
<=60	269	Reference			
> 60	270	1.765 (1.298–2.398)	**< 0.001**	2.116 (1.347–3.324)	**0.001**

### Western Blot

Proteins from each experimental group were extracted and separated by SDS-PAGE electrophoresis. Subsequently, the proteins were transferred onto PVDF membranes and blocked with 5% skim milk for 2 h. The membranes were then incubated overnight at 4 °C with the primary antibody. Following incubation, the membranes were washed with TBST and incubated with the appropriate secondary antibody. After 1 h of incubation at room temperature, the membranes were washed again with TBST and subjected to fluorescent staining.

### Cell Proliferation Assay

CCK-8 assay: 786-O cells were seeded in 96-well plates at a density of 1 × 103 cells per well and incubated overnight at 37 °C in a 5% CO2 incubator with 100 µl of 1640 medium supplemented with 10% FBS. On days 0, 1, 2, 3, and 4 post-transfection, the optical density (OD) of each well was measured at 450 nm using the Cell Counting Kit-8 (CCK-8). This assay was employed to assess the change in proliferation capacity among the different groups of cells.

EdU assay: 786-O cells were seeded in 96-well plates. Each well was incubated with 100 µl of 50 µM EdU medium for 2 h, followed by fixation with 4% formaldehyde for 30 min. Subsequently, 2 mg/ml glycine was added for 5 min, and the cells were washed with PBS for 30 min. Next, 1× ApolloR reaction mixture was added, along with EdU. Afterward, Hoechst 33,342 (400 µl) was added for 30 min, and the cells were observed under a microscope.

In summary, the CCK-8 assay was utilized to monitor the proliferation capacity of 786-O cells at different time points post-transfection. On the other hand, the EdU assay was employed to assess cell proliferation and visualize EdU incorporation in 786-O cells using fluorescence microscopy.

### Wound Healing Assay

The transfected cells and corresponding negative control cells were seeded into a six-well plate and allowed to reach confluence. A wound line was created by scratching the cell monolayer, and the wells were subsequently washed with PBS to remove any detached cells. Images of the scratch area were captured at 0 h. Following this, the cells were incubated with a low concentration serum medium to simulate cell migration. After a designated period of re-incubation, another set of images was captured to assess the migration ability of the cells.

### Transwell Assays

For the Transwell migration assay, the lower chamber of the Transwell plate was filled with 600 µl of medium containing 10% FBS. The cells, suspended in 200 µl of FBS-free medium, were seeded into the upper chamber. After 24 h of incubation, the untransfected cells were carefully removed from the upper chamber using a cotton swab. The cells that had migrated through the pores and reached the lower surface of the membrane were fixed with 4% formaldehyde. Subsequently, 400 µl of methyl violet stain was added to the cells, allowing for visualization and analysis of their invasive ability.

### Statistical Analysis

The R language (version 4.1.2) and GraphPad Prism 8.0 were used for statistical analysis. The Student’s t-test were used in the two-groupanalysis. p < 0.05 indicates statistical significance.

## Results

### TMEM44 is Differentially Expressed in Numerous Tumors and has Survival Differences Between High and Low Expression

**Fig. 1:**
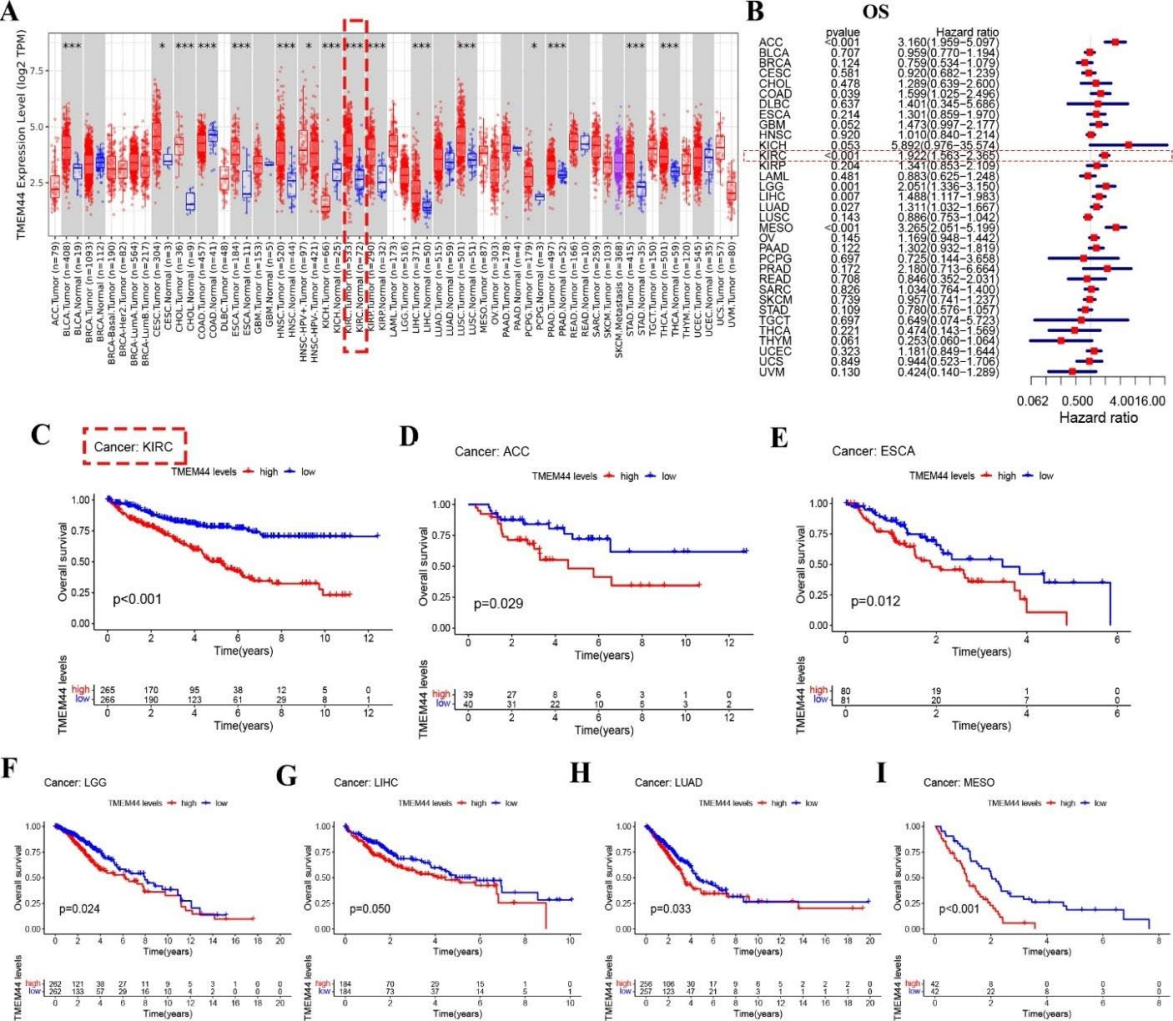
**Differential expression and overall survival analysis (COX and KM) of TMEM44 in pan-cancer**. (**A**) Pan-cancer difference analysis by TISIDB. (**B**) Overall survival analysis of pan-cancer (COX). (**C-I**) KM regression analysis of overall survival differences between high and low expression of TMEM44. (**C**) KIRC; (**D**) ACC; (**E**) ESCA; (**F**) LGG; (**G**) LIHC; (**H**) LUAD; (**I**) MESO

Analysis of the TIMER database showed significant differences in TMEM44 expression between 16 tumors and normal tissues (Fig. 1A). Moreover, after conducting overall survival (OS) analysis using COX analysis in pan-cancer, TMEM44 was identified as an independent prognostic factor in seven human tumors (ACC, COAD, KIRC, LGG, LIHC, LUAD, and MESO) (P < 0.05) (P < 0.05) (Fig. 1B). KM-plot survival analysis further demonstrated significant differences in OS between high and low expression of TMEM44 in these seven tumors (ACC, ESCA, KIRC, LGG, LIHC, LUAD, and MESO) (P < 0.05) (Fig. 1C-I).

**Fig. 2:**
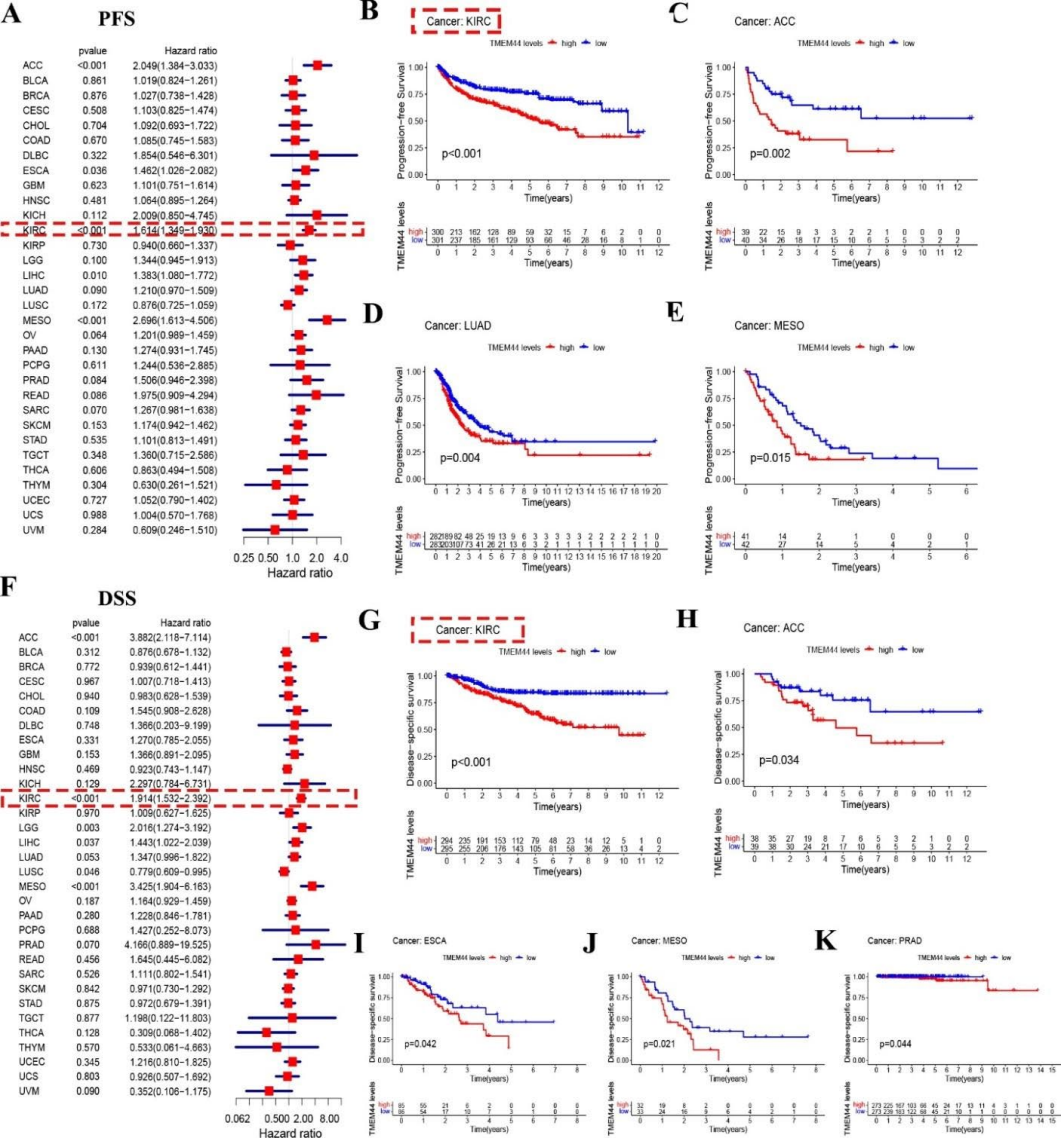
**Analysis of Progression-free survival (PFS) and Disease-specific survival (DSS) in pan-cancer**. (**A**) Progression-free survival (PFS) of pan-cancer (COX). (**B-E**) KM regression analysis of PFS differences between high and low expression of TMEM44. (**B**) KIRC; (**C**) ACC; (**D**) LUAD; (**E**) MESO. (**F**) Disease-specific survival (DSS) in pan-cancer (COX). (**G-K**) KM regression analysis of DSS differences between high and low expression of TMEM44. (**G**) KIRC; (**H**) ACC; (**I**) ESCA; (**J**) MESO; (**K**) PRAD

Pan-cancer analysis of progression-free survival (PFS) revealed that TMEM44 was an independent predictor in ACC, ESCA, KIRC, LIHC, and MESO based on COX analysis (P < 0.05) (Fig. 2A). Similarly, KM-plot survival analysis demonstrated significant differences in PFS between high and low TMEM44 expression in ACC, KIRC, LUAD, and MESO (P < 0.05) (Fig. 2B-E).

Furthermore, disease-specific survival (DSS) analysis in pan-cancer indicated that TMEM44 acted as an independent predictor in ACC, KIRC, LGG, LIHC, LUSC, and MESO based on COX analysis (P < 0.05) (Fig. 2F). KM-plot survival analysis revealed significant differences in DSS between high and low TMEM44 expression in ACC, ESCA, KIRC, MESO, and PRAD (P < 0.05) (Fig. 2G-K).

Based on the analysis of OS, PFS, and DSS of TMEM44 in pan-cancer, notable differences were observed in KIRC regarding high and low TMEM44 expression. Additionally, both COX analyses indicated that TMEM44 could serve as an independent predictor of KIRC. Therefore, we selected TMEM44 as a potential target for further investigation in KIRC.

### Clinical Correlation Analysis and Independent Prognostic Analysis

**Fig. 3:**
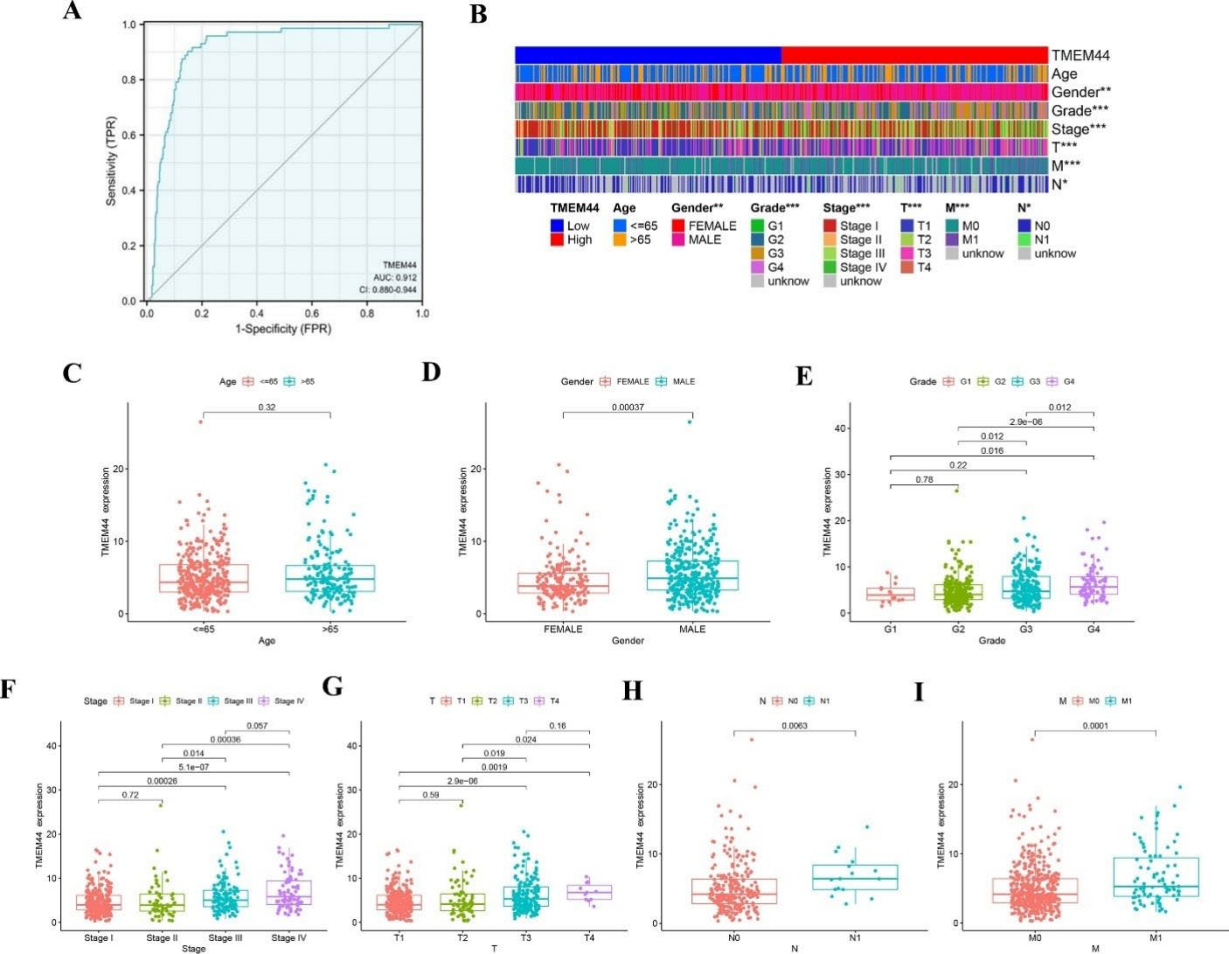
**Diagnostic value and clinical relevance of TMEM44 in KIRC**. (**A**) Diagnostic ROC curve of TMEM44 in KIRC. (**B**) Heat map of clinical data differences between high and low expression of TMEM44. (**C**) Differences in the expression of TMEM44 between different ages; (**D**) Differences in the expression of TMEM44 between the sexes; (**E**) Differences in the expression of TMEM44 between different Grade stages; (**F**) Differences in the expression of TMEM44 between different Stage stages; (**G**) Differences in the expression of TMEM44 between different T stage; (**H**) Differences in the expression of TMEM44 between different N stage; (**I**) Differences in the expression of TMEM44 between different M stages

Clinical data was extracted from the TCGA-KIRC sample, and independent prognostic analysis was conducted. The results demonstrated that TMEM44 could function as an independent prognostic factor for KIRC, as determined by combining the outcomes of univariate and multivariate COX analyses (Table 2). Diagnostic ROC curves were generated, showing an area under the curve (AUC) of 0.912 (CI: 0.880–0.944), indicating the strong predictive ability of TMEM44 (Fig. 3A). Furthermore, a heatmap analysis revealed significant differences in Age, Gender, Grade, Stage, T, M, and N between the high and low expression groups of TMEM44 (p < 0.05) (Fig. 3B). he differences in clinical traits between the high and low expression groups were visually represented using bar graphs (Fig. 3C-I).

**Table 2:** Primer sequences used for RT-Qpcr.

Gene	Sequence (5’-3’)
TMEM44	F: GGATTTGCCAAGGAAGCCAGAGAG
	R: GCAGTGTGACAGTGTGGTGAGAG
GAPDH	F: GGAGCGAGATCCCTCCAAAAT
	R: GGCTGTTGTCATACTTCTCATGG

### Construction of Nomogram and Clinical Data Validation

**Fig. 4:**
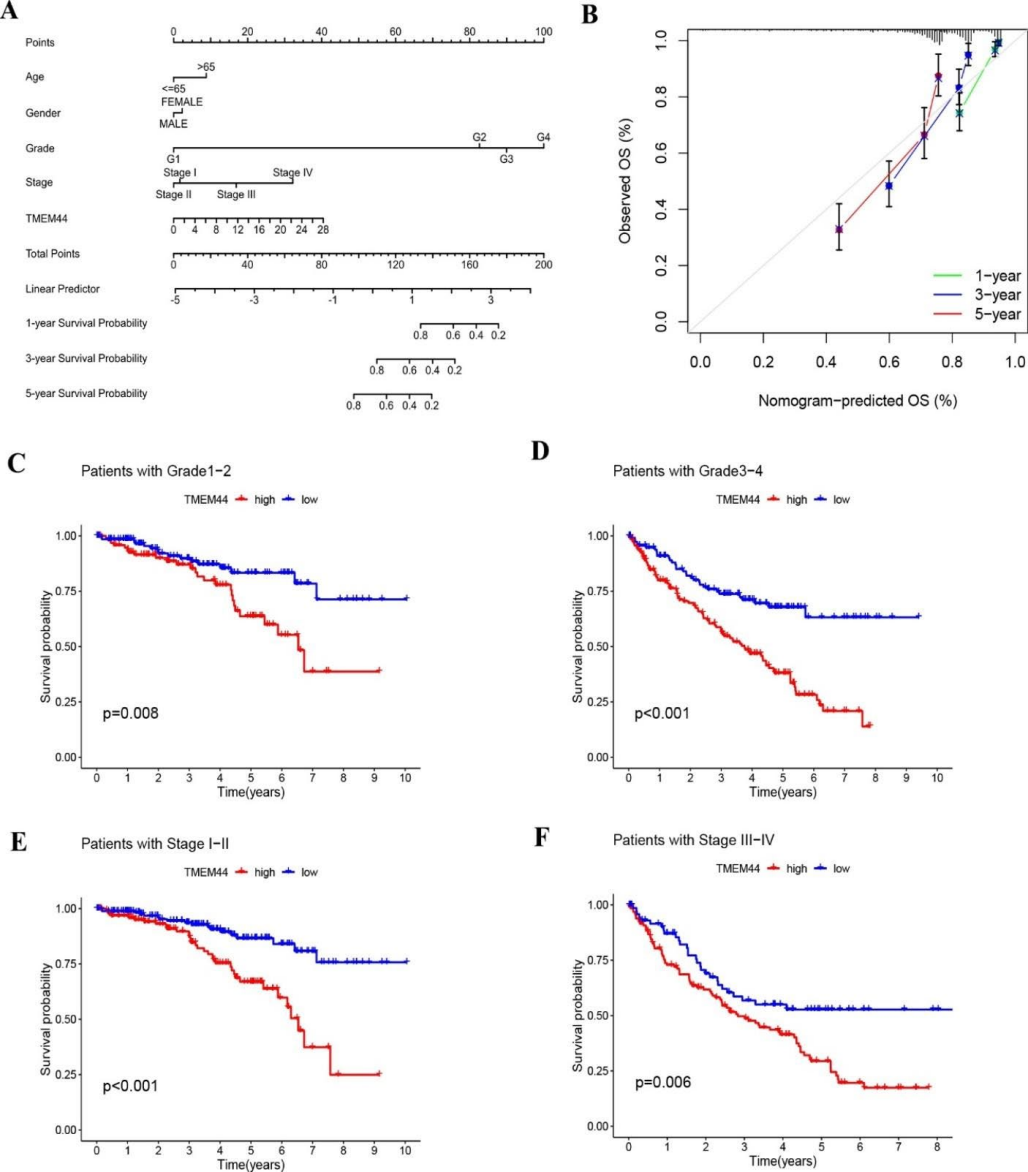
**Construction of Nomogram and Clinical Data Validation**. (**A**) Construction of Nomogram. (**B**) calibration plots of survival probabilities at 1, 3, and 5 years. (**C**) Survival analysis of patients with Grade1-2 between high and low TMEM44 expression groups. (**D**) Survival analysis of patients with Grade 3–4 between high and low TMEM44 expression groups. (**E**) Survival analysis of patients with Stage I-II between high and low TMEM44 expression groups. (**F**) Survival analysis of patients with Stage III-IV between high and low TMEM44 expression groups

Nomogram plots were constructed to enable individualized prediction of patient prognosis. Regression models were developed using TCGA-KIRC data and implemented with the “rms” package. Each indicator was assigned a score based on its contribution to the outcome variables, as determined by the magnitude of regression coefficients in each sample. These scores were then summed to calculate the total score, which was subsequently used to predict patient outcomes. Calibration plots were generated to assess the agreement between the predicted probabilities of survival at 1, 3, and 5 years and the actual observations (Fig. 4A, B). To further validate the survival difference between high and low TMEM44 expression, we divided the patients based on stage and grade into early (stage I-II and grade 1–2) and late (stage III-IV and grade 3–4) categories. Survival analysis revealed a significant difference in survival between the high and low TMEM44 expression groups in both the early and late-stage/grade patient subgroups (P < 0.05) (Fig. 4C-F).

### TMEM44 Expression was Upregulated in KIRC Tissues and Cell Lines

**Fig. 5:**
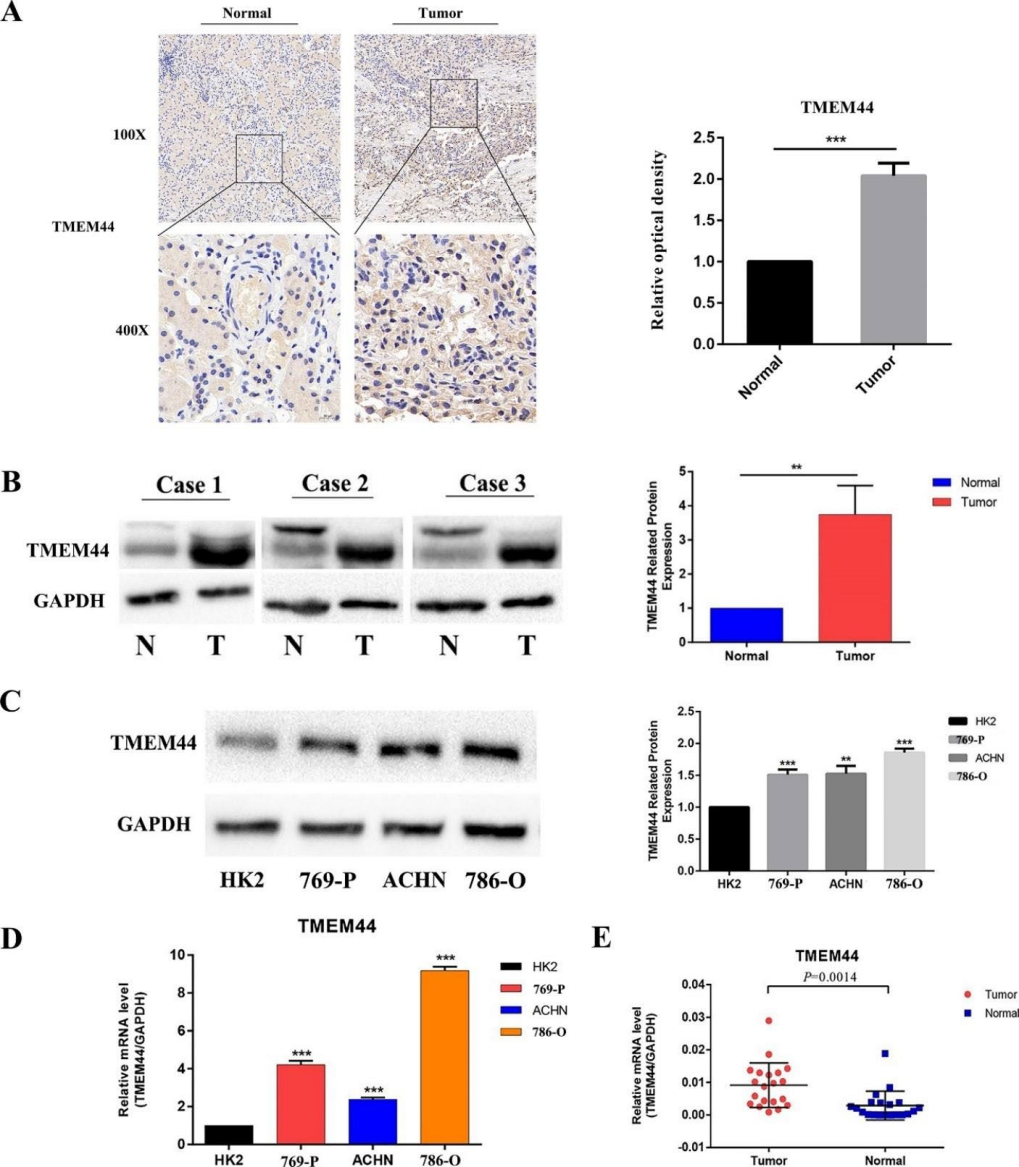
**Differential analysis of TMEM44 in KIRC tissues and cells**. (**A**) Immunohistochemical (IHC) analysis of TMEM44 in KIRC tissue and paraneoplastic tissue. (**B**) Western blot assay to analyze the difference in protein expression of TMEM44 in 3 pairs of KIRC tissues and paraneoplastic tissues. (**C**) Western blot assay to analyze the difference in protein expression of TMEM44 in KIRC cells (769-P, ACHN, 786-O) and normal cell(HK2). (**D**) q-PCR analysis of differential expression of TMEM44 in KIRC cells and normal cells. (**E**) q-PCR analysis of TMEM44 expression differences in 20 pairs of KIRC tissues and paraneoplastic tissues

To validate our analysis of TMEM44, we initially performed immunohistochemical assays to examine the protein expression levels of TMEM44 in KIRC tissues compared to paracancerous tissues. The results revealed a significant upregulation of TMEM44 in KIRC tissues (Fig. 5A). Furthermore, we conducted Western blot assays to verify the protein expression in three pairs of KIRC and adjacent tissues, which consistently showed elevated levels of TMEM44 in KIRC samples (Fig. 5B). Additionally, we assessed the protein expression of TMEM44 in three KIRC cell lines (786-O, ACHN, and 769-P) and compared it to normal kidney cells HK2. The Western blot analysis demonstrated higher abundance of TMEM44 protein in KIRC cells compared to HK2 cells (Fig. 5C). At the cellular RNA level, quantitative PCR (qPCR) assays revealed lower expression of TMEM44 in HK2 cells (Fig. 5D). Moreover, at the tissue RNA level, qPCR analysis of 20 pairs of KIRC tissues and paracancerous tissues showed increased expression of TMEM44 in KIRC tissues (Fig. 5E).

### TMEM44 Knockdown Suppressed the Proliferation and Migration of KIRC Cell Lines

**Fig. 6:**
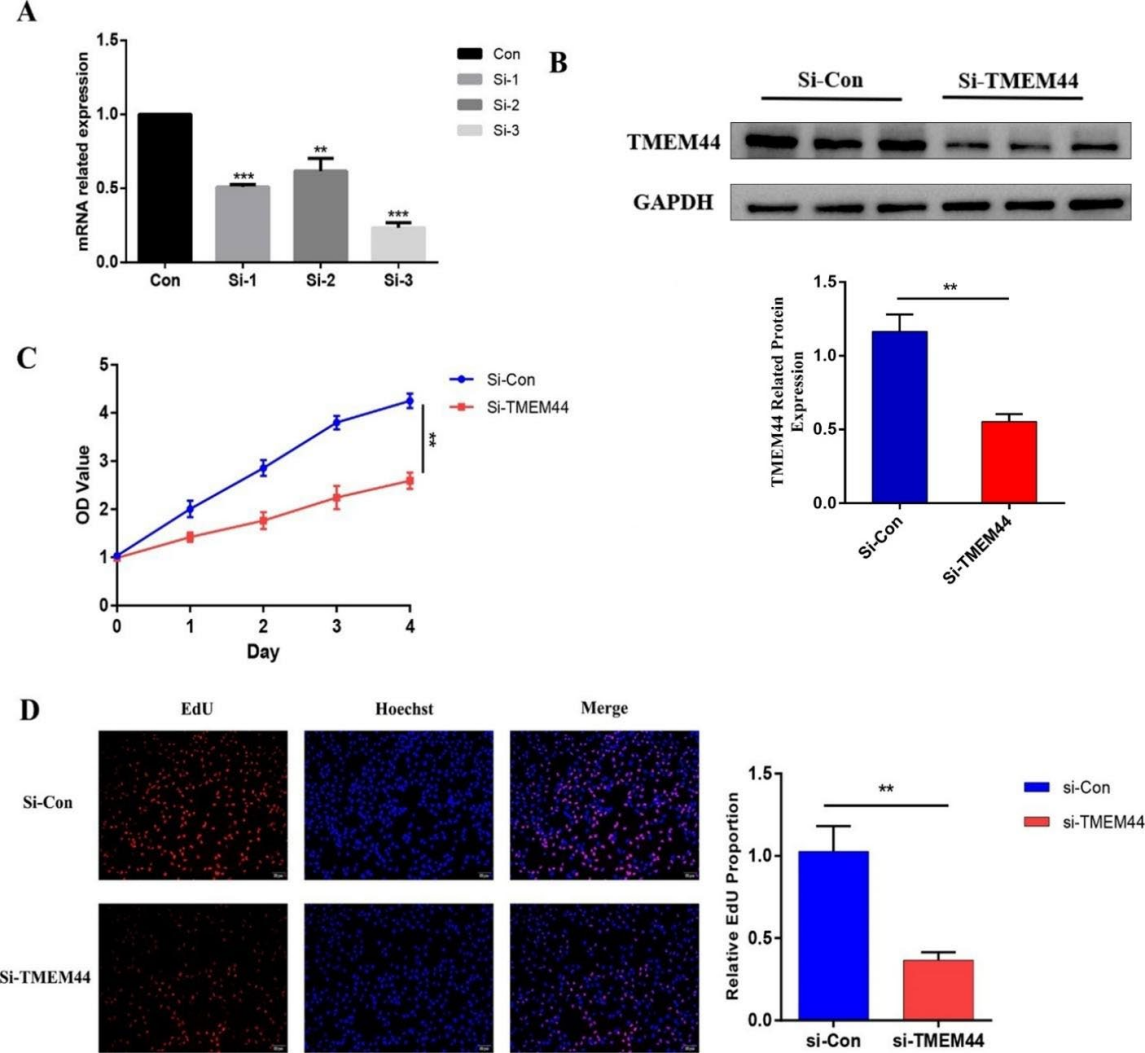
**Validation of the proliferative function of TMEM44 after knockdown**. (**A**) q-PCR validates the efficiency of three inhibitors to knock down TMEM44. (**B**) Western blot assay to analyze the difference in protein expression after TMEM44 knockdown. (**C**) CCK-8 experiments to analyze the proliferation between si-TMEM44 and si-Con. (**D**) EdU experiments to analyze the proliferation between si-TMEM44 and si-con

To validate TMEM44’s role in KIRC, we selected 786-O cells for TMEM44 expression intervention in functional validation experiments. Three siRNAs (si-1, si-2, and si-3) were designed based on TMEM44 gene sequences and transfected into 786-O cells, while si-control was transfected into the control group. The inhibitory efficiency of the three siRNAs was evaluated using qPCR. The results indicated that si-3 had the most effective inhibitory effect, thus si-3 (referred to as si-TMEM44 in the figures) was selected for further experiments (Fig. 6A). Subsequently, Western blot analysis confirmed a significant reduction in TMEM44 protein expression in the si-TMEM44 group compared to the si-control group (Fig. 6B).

**Fig. 7:**
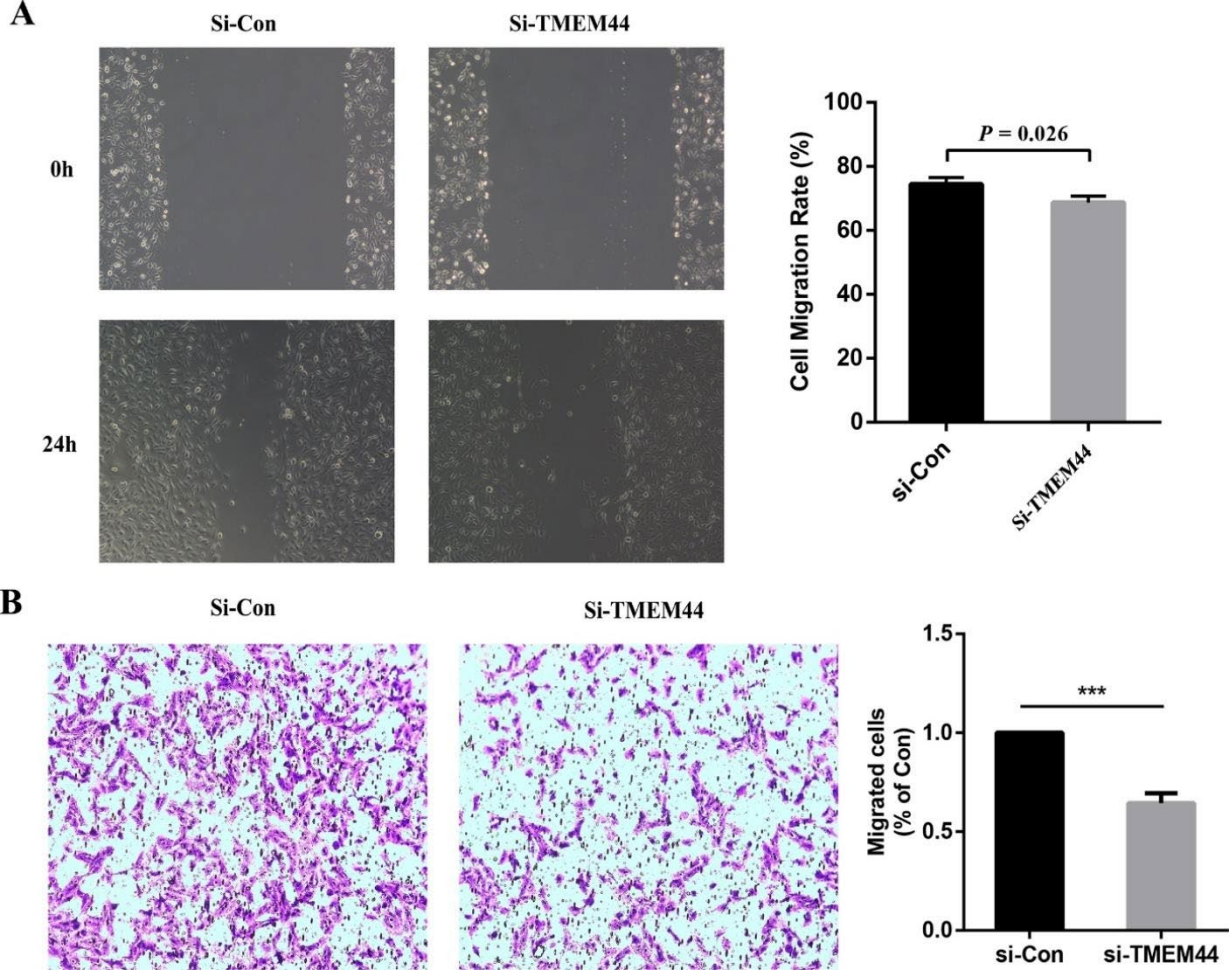
**Cell scratch assay and Transwell assay**. (**A**) Cell migration ability of si-Con and si-TMEM44 analyzed by cell scratching assay. (**B**) Transwell analysis of cell migration ability of si-Con and si-TMEM44

To assess the impact of TMEM44 knockdown on cell proliferation, a CCK-8 assay was performed. The results demonstrated that si-TMEM44 significantly inhibited the proliferation of 786-O cells (Fig. 6C). The EdU assay further confirmed that downregulation of TMEM44 expression in 786-O cells significantly affected tumor cell proliferation (Fig. 6D). Moreover, wound healing and Transwell assays were conducted to evaluate cell migration ability. The results showed a significant reduction in migrating cells in the si-TMEM44 group compared to the control group (Fig. 7A, B). These findings indicate that silencing TMEM44 suppressed the proliferation and migration abilities of KIRC cells.

To further enhance the reliability of the experiment, another validation was performed using the si-1 interference fragment. Initially, the interference efficiency of si-1 at the protein level was confirmed (Figure S1A). Consistently, CCK-8 and EdU experiments verified that knocking down TMEM44 expression using si-1 resulted in a significant reduction in the proliferation ability of KIRC cells (Figure S1B, C). Additionally, Transwell and wound healing assays were repeated after TMEM44 knockdown using si-1, and the results were consistent with the previous findings, demonstrating the effective inhibition of migratory ability in KIRC cells (Figure S2).

## Discussion

TMEM44, a member of the TMEM family, has been relatively understudied. However, research has revealed that its antisense RNA, TMEM44-AS1, can promote the growth, migration, and invasion of glioma cells. It forms a positive feedback loop with Myc/MED1, affecting tumor development in glioma^(Bian et al. 2021)^. Additionally, overexpression of VSX1 has been shown to increase the activity of TMEM44, FKBP10, and TRIB3, and enhance the growth of renal clear cell carcinoma^(Ma et al. 2022)^. Therefore, investigating whether VSX1 influences the progression of renal clear cell carcinoma through downstream TMEM44 warrants further investigation. Moreover, the use of a novel nanocarrier called chitosan-gelatin-EGCG (CGE) has been shown to effectively silence TMEM44-AS1 expression and reverse 5-FU resistance in gastric cancer, highlighting the value of studying TMEM44 antisense RNA in tumors^(Zhou et al. 2022)^. These findings have sparked interest in investigating the influence of TMEM44 on tumor development.

In our study of TMEM family genes, we found that TMEMs have diverse roles in tumors. Firstly, some TMEMs act as tumor suppressors. For example, TMEM25 affects the development of colon cancer as a tumor suppressor^(Hrašovec et al. 2013)^. Secondly, many TMEMs act as oncogenes, being upregulated in tumors and associated with tumor progression, invasion, and metastasis formation. These TMEMs can serve as prognostic biomarkers. TMEM45A, for instance, is overexpressed in various cancers, including ovarian, kidney, and liver cancers^(Flamant et al. 2012; Jiang et al. 2021; Guo et al. 2015)^. Thirdly, TMEMs are involved in chemoresistance. TMEM45A has been closely associated with chemoresistance in hepatocellular carcinoma^(Jiang et al. 2021)^. TMEM88 has also been found to be associated with increased resistance to platinum in ovarian cancer^(Leon et al. 2016)^. Thus, the role of TMEM44 in tumors is of particular interest.

In our study, we initially screened the prognostic marker role of TMEM44 in KIRC through pan-cancer survival analysis, and further investigation confirmed its significance. We demonstrated that knockdown of TMEM44 effectively slowed down the proliferation and migration of cancer cells, consistent with our bioinformatics analysis. Moreover, analysis of online database data revealed that TMEM44 serves not only as a prognostic biomarker for KIRC but also plays a role in guiding immunotherapy. The expression of TMEM44 in KIRC can distinguish the abundance of several immune cells, which is relevant to immunotherapy. As immune checkpoint inhibitors are a hot topic in tumor therapy, our correlation analysis of TMEM44 with selected immune checkpoints showed significant associations. Furthermore, our analysis of the risk of immunotherapy resistance indicated that the high-risk group may have a worse response to immunotherapy. These findings provide valuable insights for the treatment and prognosis of KIRC.

Although the function of TMEM44 remains poorly understood, our study and bioinformatics analysis suggest that TMEM44 can significantly influence tumor progression in KIRC and is an important member of the TMEM family. Furthermore, the role of TMEM44 in immune cells and its potential influence on resistance to chemotherapeutic agents in KIRC warrant further investigation. One limitation of this study is that we were unable to elucidate the underlying mechanism by which TMEM44 affects tumor progression. Therefore, further in-depth studies in this direction are warranted.

## Conclusion

In conclusion, our study has provided compelling evidence regarding the significance of TMEM44 as an independent prognostic factor in KIRC patients, establishing its potential as a valuable prognostic marker for this disease. Furthermore, through a comprehensive series of in vitro functional assays, we have elucidated the role of TMEM44 in promoting tumor development in KIRC. This discovery not only enhances our understanding of the underlying mechanisms driving KIRC progression but also offers a novel therapeutic target for the treatment of this aggressive cancer. Importantly, our findings shed light on the potential of targeting TMEM44 for immunotherapy in KIRC patients, opening up promising avenues for future research and the development of more effective treatment strategies.

### Electronic Supplementary Material

Below is the link to the electronic supplementary material.

Supplementary Material 1

Supplementary Material 2

Supplementary Material 3

## References

[CR11] Bian E (2021). Super-enhancer-associated TMEM44-AS1 aggravated glioma progression by forming a positive feedback loop with myc. J Experimental Clin Cancer Research: CR.

[CR17] de Leon M (2016). Transmembrane protein 88 (TMEM88) promoter hypomethylation is associated with platinum resistance in ovarian cancer. Gynecol Oncol.

[CR9] Duan J (2021). TMEM106C contributes to the malignant characteristics and poor prognosis of hepatocellular carcinoma. Aging.

[CR14] Flamant L (2012). TMEM45A is essential for hypoxia-induced chemoresistance in breast and liver cancer cells. BMC Cancer.

[CR5] Foulquier F (2012). TMEM165 deficiency causes a congenital disorder of glycosylation. Am J Hum Genet.

[CR16] Guo J (2015). Inhibition of TMEM45A suppresses proliferation, induces cell cycle arrest and reduces cell invasion in human ovarian cancer cells. Oncol Rep.

[CR13] Hrašovec S et al (2013) *TMEM25 is a candidate biomarker methylated and down-regulated in colorectal cancer*. Dis Markers, 34(2) 10.3233/DMA-120948 PMC3809969 23324576

[CR15] Jiang H (2021). Upregulation of TMEM45A promoted the progression of Clear Cell Renal Cell Carcinoma in vitro. J Inflamm Res.

[CR12] Ma W (2022). High VSX1 expression promotes the aggressiveness of clear cell renal cell carcinoma by transcriptionally regulating FKBP10. J Translational Med.

[CR8] Rao J (2020). TMEM205 is an independent prognostic factor and is Associated with Immune Cell infiltrates in Hepatocellular Carcinoma. Front Genet.

[CR1] Schmit K, Michiels C (2018). TMEM Proteins in Cancer: a review. Front Pharmacol.

[CR7] Shen K et al (2018) Knockdown of TMEM45B inhibits cell proliferation and invasion in gastric cancer, vol 104. Biomedicine & Pharmacotherapy = Biomedecine & Pharmacotherapie, pp 576–581 10.1016/j.biopha.2018.05.016 29803169

[CR6] Thomas-Gatewood C (2011). TMEM16A channels generate Ca^2+^-activated Cl^−^ currents in cerebral artery smooth muscle cells. Am J Physiol Heart Circ Physiol.

[CR2] Vinothkumar KR, Henderson R (2010) *Structures of membrane proteins*. Q Rev Biophys, 43(1) 10.1017/S0033583510000041 PMC3604715 20667175

[CR3] von Heijne G (2006). Membrane-protein topology. Nat Rev Mol Cell Biol.

[CR4] Zhao H et al (2017) TMEM88 inhibits extracellular matrix expression in keloid fibroblasts, vol 95. Biomedicine & Pharmacotherapy = Biomedecine & Pharmacotherapie, pp 1436–1440 10.1016/j.biopha.2017.09.047 28946191

[CR10] Zhou M (2022). Chitosan-Gelatin-EGCG nanoparticle-meditated LncRNA TMEM44-AS1 silencing to activate the P53 signaling pathway for the synergistic reversal of 5-FU resistance in gastric Cancer. Adv Sci (Weinheim Baden-Wurttemberg Germany).

