# Supplementary figures and images for: TMEM44 as a Novel Prognostic Marker for Kidney Renal Clear Cell Carcinoma is Associated with Tumor Invasion, Migration and Immune Infiltration
Source: Biochem Genet. 2023 Aug 10;62(2):1200–15. doi: 10.1007/s10528-023-10466-x (PMC11031452; doi:10.1007/s10528-023-10466-x)

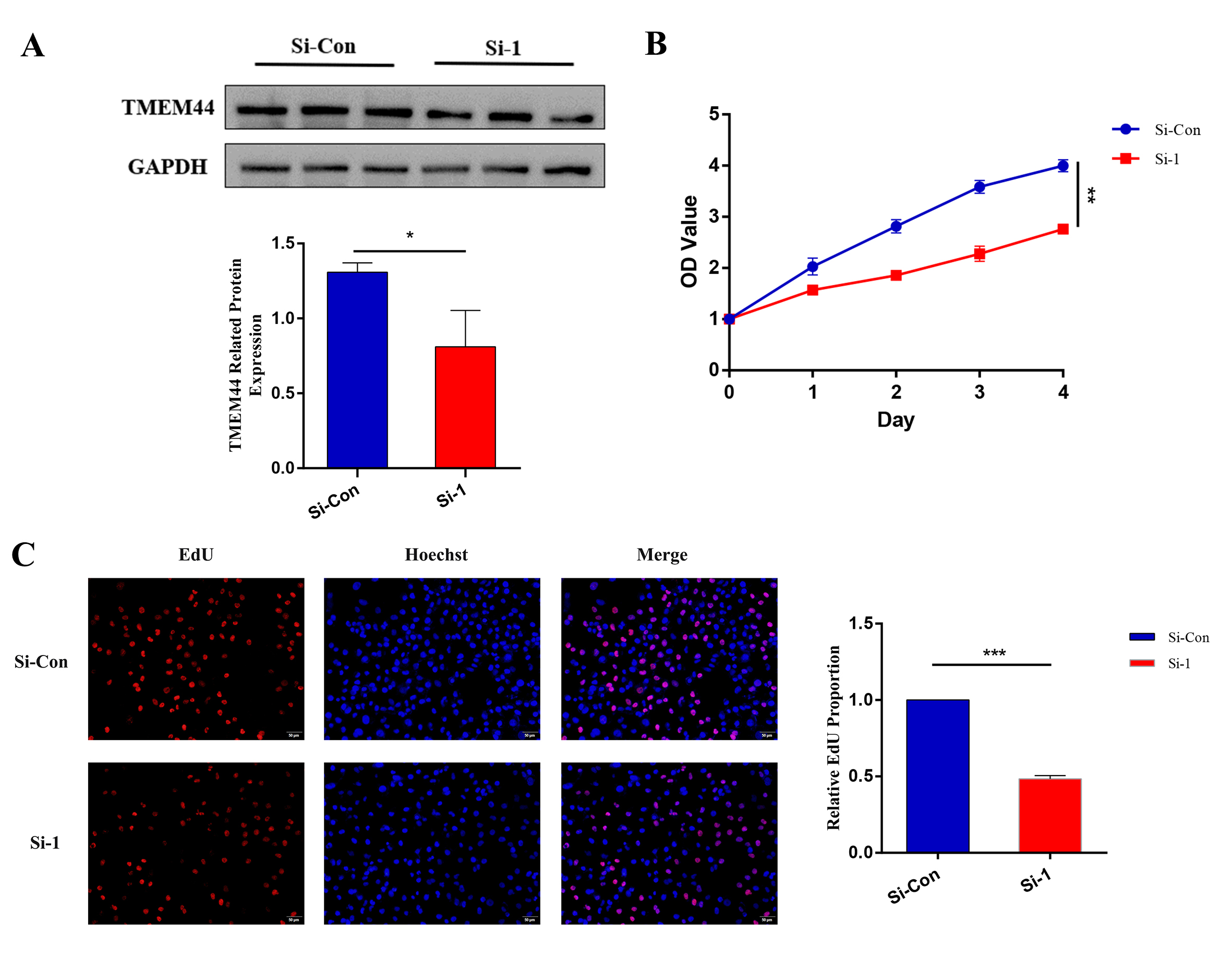

Supplement: Supplementary file 1 — Supplementary Material 1 [file 10528_2023_10466_MOESM1_ESM.jpg]

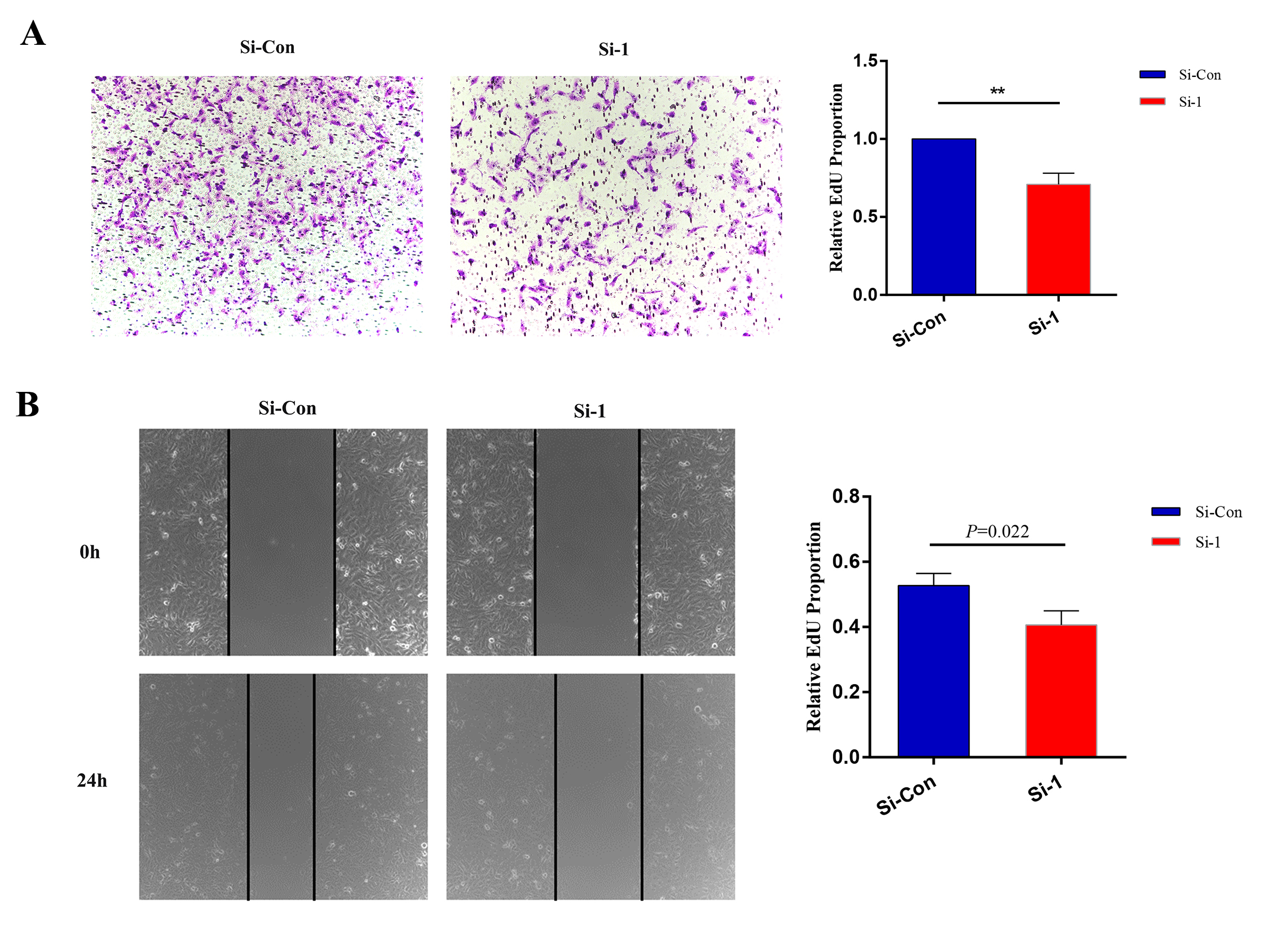

Supplement: Supplementary file 2 — Supplementary Material 2 [file 10528_2023_10466_MOESM2_ESM.jpg]
