# Supplementary material for: TMEM44 as a Novel Prognostic Marker for Kidney Renal Clear Cell Carcinoma is Associated with Tumor Invasion, Migration and Immune Infiltration
Source: Biochem Genet. 2023 Aug 10;62(2):1200–15. doi: 10.1007/s10528-023-10466-x (PMC11031452; doi:10.1007/s10528-023-10466-x)
Supplement: Supplementary file 3 — Supplementary Material 3 [file 10528_2023_10466_MOESM3_ESM.docx]

**Table S1 | Interference fragment sequence of TMEM44**

| Gene | sense (5’-3’) | Antisense (5’-3’) |
| --- | --- | --- |
| Si-1 | CCUAGCAGCUAUUGACUUATT | UAAGUCAAUAGCUGCUAGGTT |
| Si-2 | GCACUGGACCUCGCUAUUATT | UAAUAGCGAGGUCCAGUGCTT |
| Si-3 | GGGACCCUGAAGAUGUGAATT | UUCACAUCUUCAGGGUCCCTT |
